# Genotype and Phenotype of 198 Korean Patients With Nonlethal Osteogenesis Imperfecta: A Retrospective Cohort Study

**DOI:** 10.1155/humu/5521427

**Published:** 2026-09-27

**Authors:** Junpyo Lee, Hye Ran Lee, Jung Min Ko, Chang Ho Shin, Wonik Lee, Mi Hyun Song, Tae-Joon Cho

**Affiliations:** ^1^ Division of Pediatric Orthopaedics, Seoul National University Children′s Hospital, Department of Orthopaedics, Seoul National University College of Medicine, Seoul, South Korea, snuh.org; ^2^ Department of Pediatrics, Seoul National University College of Medicine, Seoul, South Korea, snuh.org

## Abstract

Osteogenesis imperfecta (OI) is a heterogeneous group of genetic skeletal disorders characterized by bone fragility and low bone mass, manifesting as multiple fractures and deformities of the spine and extremities. Although genotype‐phenotype correlations have been studied in various populations, large‐scale data in Koreans have been lacking. This study is aimed at characterizing the clinical and genetic spectrum of nonlethal OI in the Korean population and explore genotype–phenotype associations. This retrospective cohort study analyzed 198 genetically confirmed Korean OI probands evaluated at a single center between 2004 and 2024, with clinical features reviewed and genetic testing performed using Sanger sequencing and targeted next‐generation sequencing. A total of 160 distinct pathogenic variants were identified, including 44 novel variants. Variant pathogenicity was assessed according to the ACMG/AMP variant classification guidelines. Pathogenic variants of *COL1A1* and *COL1A2* accounted for 87.4% of cases, with *COL1A1* variants being the most prevalent (66.7%). Only 4.0% of cases had autosomal recessive OI. Glycine substitution (GS) variants were associated with significantly more severe phenotypes than other variants, including shorter height (median z‐score−3.67 [IQR −8.37 to −1.78] vs. −1.17 [−1.96 to −0.16], *p* < 0.001) and earlier fracture onset (1.00 [0.00–8.25] vs. 15.00 [7.00–24.75] months, *p* < 0.001). In *COL1A1*, GS variants closer to the C‐terminus correlated with significantly more severe height reduction (Spearman *ρ* = −0.478, *p* = 0.005). The genotype–phenotype analysis confirmed the phenotypic severity of GS, particularly for variants closer to the C‐terminus of *COL1A1*, consistent with previous studies. Additionally, the identification of 44 novel variants expands the variant spectrum of OI. These findings provide valuable insights into genetic diagnosis, prognostic assessment, and clinical management of OI.

## 1. Introduction

Osteogenesis imperfecta (OI) is a heterogeneous group of genetic skeletal disorders characterized by bone fragility and low bone mass, manifesting as multiple fractures and deformities of the spine and extremities [1].

The modified Sillence classification system is based on clinical severity categorizing the patients into types 1–5 [2]. In addition to this clinical classification, a molecular classification system based on causative genes has also been proposed [3].

With advances in genetic studies, 25 causative genes have been identified to date, and these genes are involved in diverse biological pathways [4, 5]. OI can be inherited in an autosomal dominant (AD), autosomal recessive (AR), or X‐linked manner [3].

The major structural protein of the extracellular matrix in bone, tendon, and skin is Type I collagen, composed of two *α*1 chains and one *α*2 chain, encoded by the *COL1A1* and *COL1A2* genes, respectively [6]. These chains are composed of an N‐terminal region, a C‐terminal region, and a central triple helical domain. The triple helical domain is composed of repeating Gly‐X‐Y tripeptides, where glycine plays a crucial role in maintaining the proper folding and stability of the collagen helical structure [7]. Variants in *COL1A1* or *COL1A2* can disrupt this structure, particularly glycine substitution (GS) variants, which interfere with triple helix formation and result in the clinical features of OI [8].

Genetic studies on OI have been conducted across various populations, including Canadian, Swedish, Japanese, and Chinese cohorts [9–13]. Along with these studies, additional population‐based research has demonstrated that the distribution of causative genes varies across ethnic groups and countries, reflecting underlying genetic diversity [14, 15]. In addition to identifying causative genes, many of these studies have also examined genotype‐phenotype correlations, particularly focusing on GS variants in the *COL1A1* and *COL1A2* genes. It has been shown that both the location of the GS within the triple helical domain and the nature of the substituting amino acid significantly influence the severity and clinical characteristics of OI phenotypes [9, 11]. However, a large‐scale study in the Korean population has not yet been conducted.

In this study, we aimed to analyze the clinical characteristics and genotype–phenotype correlations in patients with nonlethal OI in the Korean population. Additionally, we sought to identify and characterize novel variants in OI‐related genes that have not been previously reported in existing databases, thereby contributing to the understanding of the genetic and phenotypic diversity of OI in this population.

## 2. Materials and Methods

### 2.1. Subject

This study was approved by the Institutional Review Board of Seoul National University Hospital. We retrospectively reviewed patients with nonlethal OI treated at Seoul National University Children′s Hospital between Oct 2004 and May 2024 who had confirmed pathogenic gene variants. The perinatal lethal form (Sillence Type 2) was not included. Of 279 patients initially identified, 19 individuals with incomplete medical records were excluded. Among the remaining patients, 62 were excluded so that only one proband with the most comprehensive and well‐documented clinical history per family was included. In total, 198 probands were analyzed. All individuals were of Korean ethnicity, minimizing the risk of population stratification due to ethnic heterogeneity.

### 2.2. Clinical Analysis

Clinical data were retrospectively obtained from medical chart review including physical phenotype, fracture history, anthropometric measures and serial radiographs. Sillence type was classified by a pediatric orthopedic surgeon based on clinical and radiographic findings [2].

During follow‐ups, additional fractures, growth, scoliosis, and mobility status were updated. Clinical variables were assessed at the latest available follow‐up, and that age was designated as the age at the latest follow‐up. However, height, weight, and BMI were not measured at every visit; the age at the last available anthropometric measurement was separately recorded as the age of measurement. The z‐score of height, weight, and BMI was calculated according to age and sex using the 2017 Korean Pediatric Growth Standards from the Korean Disease Control and Prevention Agency [16].

The initial fracture age was defined as the earliest documented fracture, recorded in months, with birth fractures or perinatal deformities assigned as 0 months. Fracture number was categorized as ≤ 10 or > 10. However, in very young patients, absolute fracture counts may underestimate clinical severity. Because fracture incidence peaks during the growing years, skeletal maturity was considered in the analysis [17]. To balance accuracy with sample size, age 14 was chosen as practical threshold, reflecting the average age of skeletal maturity in females [18], and only patients older than 14 years were included fracture number analysis.

Although bisphosphonate therapy may influence fracture risk [19], a detailed analysis based on treatment timing was not feasible. However, to assess whether bisphosphonate treatment was clinically indicated, treatment status was documented for each patient.

Wormian bone was defined as more than 10 bone fragments [20], and scoliosis as a Cobb angle > 25° on the latest radiograph [21]. Mobility status was categorized into three groups based on outdoor ambulatory status: walk without aid, walk with aid, and wheelchair bound.

Because the data were obtained retrospectively, some variables were missing. Analyses were conducted using available data, with sample sizes (*n*) specified in the tables.

### 2.3. Genetic Analysis

Genetic testing evolved over the study period. From 2004 to 2012, Sanger sequencing of *COL1A1, COL1A2, IFITM5*, and *FKBP10* was performed [22]. Patients from this period in whom no pathogenic variant was identified were subsequently reanalyzed in 2013 by targeted exome sequencing (TES) of 255 skeletal dysplasia–associated genes [23]. From 2013, TES also served as the first‐line genetic test for newly evaluated patients, with the panel subsequently expanded to 268 genes in 2014 and 346 in 2015. From 2016, focused exome sequencing (Agilent SureSelect Focused Exome, S07084713; Agilent Technologies, Santa Clara, California, United States) became the primary approach, and a 24‐gene clinical OI panel was additionally used from 2021. Patients in whom no pathogenic variant was identified by any of the preceding methods were finally analyzed by whole‐exome sequencing (Agilent SureSelect Human All Exon V5, S04380110; Agilent Technologies, Santa Clara, California, United States). Pathogenic variants were confirmed by Sanger sequencing, with familial segregation analysis when available. The gene content of each targeted panel is provided in Table S1.

Novel variants were identified by their absence from the OI and Ehlers–Danlos Syndrome Variant Database (https://collagen.le.ac.uk/), the Human Gene Mutation Database (https://www.hgmd.cf.ac.uk/ac/index.php), and ClinVar (http://www.ncbi.nlm.nih.gov/clinvar/). These variants were interpreted and classified based on the criteria established by the 2015 guidelines of the American College of Medical Genetics and Genomics and the Association for Molecular Pathology (ACMG/AMP) [24]. Variant interpretation included segregation analysis when available, supported by InterVar (https://wintervar.wglab.org/), a bioinformatics tool for clinical classification of genetic variants [25], and review of allele frequencies from population databases such as KOVA (Korean Variant Archive for a reference database of genetic variations in the Korean population), (https://kobic.re.kr/kova/) [26], gnomAD (https://gnomad.broadinstitute.org/), NCBI dbSNP (https://www.ncbi.nlm.nih.gov/snp), and Ensembl (https://asia.ensembl.org).

### 2.4. Statistical Analysis

Statistical analyses were performed using SPSS Version 25 (IBM Corp.) and Python Version 3.13. Normality and homogeneity of variance were assessed using the Shapiro–Wilk and Levene′s tests, respectively. Nonnormally distributed variables (height z‐score, weight z‐score, and initial fracture age) are presented as median (interquartile range, IQR), normally distributed variables (e.g., age, BMI z‐score) as mean ± standard deviation, and categorical variables as *n* (%). For comparisons among Sillence Types 1, 3, and 4 (Type 5 was described but not compared), nonnormal variables were analyzed by the Kruskal–Wallis test with pairwise Mann–Whitney *U* tests (Bonferroni‐adjusted *p* values, the raw *p* value multiplied by the number of comparisons and capped at 1.0), normal variables by one‐way ANOVA (or Welch′s ANOVA when variances were unequal), and categorical variables by the chi‐square or Fisher′s exact test. For comparisons between the GS and other variants (OV) groups in *COL1A1*/*COL1A2*, nonnormal variables were analyzed by the Mann–Whitney *U* test, normal variables by the independent‐samples *t*‐test, and categorical variables by the chi‐square or Fisher′s exact test. Odds ratios were corrected using the Haldane–Anscombe method when a zero cell was present. A two‐sided *p* < 0.05 was considered statistically significant.

Height z‐score, a clinically relevant marker of OI severity, was plotted against the GS locus (triple‐helical residue position on the *x*‐axis, height z‐score on the *y*‐axis) separately for *COL1A1* and *COL1A2*. Because the association between variant position and height was expected to be monotonic rather than strictly linear, and the variables were nonnormally distributed, Spearman′s rank correlation coefficient was used to assess the association.

Patients with substantially incomplete clinical records were excluded during cohort selection. Individuals with minor missing variables were included but excluded from analyses involving those specific variables, with no data imputation. This study followed the STREGA (STrengthening the REporting of Genetic Association Studies) extension of the STROBE Statement (checklist provided as Supporting Information) [27].

## 3. Results

### 3.1. Patient Characteristics

A total of 198 genetically confirmed OI probands (106 males and 92 females) were included. The distribution of Sillence types was Type 1 (108, 54.5%), Type 3 (32, 16.2%), Type 4 (42, 21.2%), and Type 5 (16, 8.1%) (Table 1). A family history was most prevalent in Type 1 (60.0%) and significantly less frequent in Types 3 and 4.

**Table 1:** Characteristics of the study population.

	Modified Sillence classification			
	Type 1 (*N* = 108)	Type 3 (*N* = 32)	Type 4 (*N* = 42)	Type 5 (*N* = 16)
Sex				
Male/female	58/50	15/17	25/17	8/8
Family history				
Positive/total	60/100 (60.0%)^†††^	4/23 (17.4%) ^∗^	14/40 (35.0%)	6/16 (37.5%)
Age at the latest follow‐up				
Years, mean ± SD	17.16 ± 10.74	26.11 ± 13.30^∗^	22.01 ± 11.03	24.29 ± 14.28
	(*N* = 108)	(*N* = 32)	(*N* = 42)	(*N* = 16)
Age of measurement				
Years, mean ± SD	14.50 ± 9.14	22.29 ± 12.48^∗∗∗^	18.64 ± 9.96	19.50 ± 13.60
Height (z‐score)				
Median [IQR]	−1.15 [−1.79 to −0.17]	−9.04 [−14.20 to −5.90] ^∗^	−2.90 [−6.02 to −2.07] ^∗^^‡^	−2.03 [−2.72 to −0.75]
	(*N* = 100)	(*N* = 28)	(*N* = 41)	(*N* = 13)
Weight (z‐score)				
Median [IQR]	−0.44 [−1.07 to 0.71]	−1.40 [−5.14 to −1.28] ^∗^	−0.62 [−1.29 to 0.28]^‡^	−0.76 [−2.22 to 0.11]
	(*N* = 81)	(*N* = 28)	(*N* = 39)	(*N* = 14)
BMI (z‐score)				
Mean ± SD	0.34 ± 1.63	1.27 ± 2.57	0.96 ± 2.09	0.41 ± 2.06
	(*N* = 80)	(*N* = 27)	(*N* = 37)	(*N* = 13)
Initial fracture age				
Months, median [IQR]	15.00 [8.00–30.00]	0.00 [0.00–0.15] ^∗^	1.00 [0.00–8.75] ^∗^^‡‡^	2.00 [0.38–7.00]
	(*N* = 89)	(*N* = 18)	(*N* = 38)	(*N* = 11)
Fracture No. (age > 14 years)				
≤ 10	38 (65.5%)^†††^	0 (0.0%) ^∗^	10 (33.3%)^‡‡‡^	2 (16.7%)
> 10	20 (34.5%)	17 (100.0%)	20 (66.7%)	10 (83.3%)
Bisphosphonate Tx.				
Positive/total	60/102 (58.8%)	18/19 (94.7%) ^∗∗∗^	35/38 (92.1%) ^∗∗^	11/13 (84.6%)
Dentinogenesis imperfecta				
Positive/total	29/82 (35.4%)	12/15 (80.0%) ^∗∗^	20/33 (60.6%)	0/11 (0.0%)
Wormian bone				
Positive/total	40/82 (48.8%)	14/16 (87.5%) ^∗∗∗^	29/39 (74.4%) ^∗∗∗^	12/13 (92.3%)
Scoliosis				
Positive/total	6/100 (6.0%)	25/29 (86.2%) ^∗^^†^	14/40 (35.0%) ^∗^	10/16 (62.5%)
Mobility status				
Walk without aid	96 (94.1%)†	0 (0.0%) ^∗^^†^	19 (48.7%)	8 (53.3%)
Walk with aid	4 (3.9%)	9 (31.0%)	17 (43.6%) ^∗^	3 (20.0%)
Wheelchair‐bound	2 (2.0%)	20 (69.0%)	3 (7.7%)	4 (26.7%)

*Note:* Age of measurement refers to the age at which height, weight, and BMI were recorded. Superscripts denote pairwise differences based on Bonferroni‐adjusted *p* values (raw p × 3, capped at 1.0); ∗*p* < 0.001, ∗∗*p* < 0.01, ∗∗∗*p* < 0.05 versus Type 1; †*p* < 0.001, ††*p* < 0.01, †††*p* < 0.05 versus Type 4; ‡*p* < 0.001, ‡‡*p* < 0.01, ‡‡‡*p* < 0.05 versus Type 3.

Abbreviations: BMI, body mass index; IQR, interquartile range; SD, standard deviation; Tx., treatment.

Clinical severity differed significantly across Sillence Types 1, 3, and 4 (Table 1). Height was most severely affected in type 3 (median z‐score−9.04 [IQR −14.20 to −5.90]), followed by Type 4 (−2.90 [−6.02 to −2.07]) and Type 1 (−1.15 [−1.79 to −0.17]), with all pairwise differences statistically significant. Consistent with this gradient, patients with Types 3 and 4 also showed earlier fracture onset, a greater fracture burden, and more frequent bisphosphonate treatment, scoliosis, and mobility impairment than Type 1, with Type 3 generally being the most severe (Table 1).

### 3.2. Genotype of Study Subjects

Genetic analysis of the 198 OI probands revealed pathogenic variants most frequently in *COL1A1* (132 probands, 66.7%), followed by *COL1A2* (41, 20.7%), *IFITM5* (17, 8.6%), *FKBP10* (6, 3.0%), and single cases in *BMP1* and *WNT1*. Although these proportions are based on probands, 84 of 190 AD OI probands had one or more affected parents or siblings, whereas all autosomal recessive osteogenesis imperfecta (AR OI) cases occurred in sporadic families. Among all identified pathogenic variants, 19 variants were found in at least two probands, and these variants were identified in 64 independent probands (Table S2). The most frequent was *IFITM5* c.−14C>T, observed in 16 probands and classified as a regulatory variant.

Overall, these included distinct variants in *COL1A1* (112 variants), *COL1A2* (34), *IFITM5* (2), *FKBP10* (8), *BMP1* (2), and *WNT1* (2) (Figure 1A). Regarding variant types, substitutions were the most common (*n* = 112), followed by deletions (*n* = 34) (Figure 1B). In terms of variant effect, missense variants were the most common (*n* = 64), followed by frameshift variants (*n* = 42) (Figure 1C).

**Figure 1:**
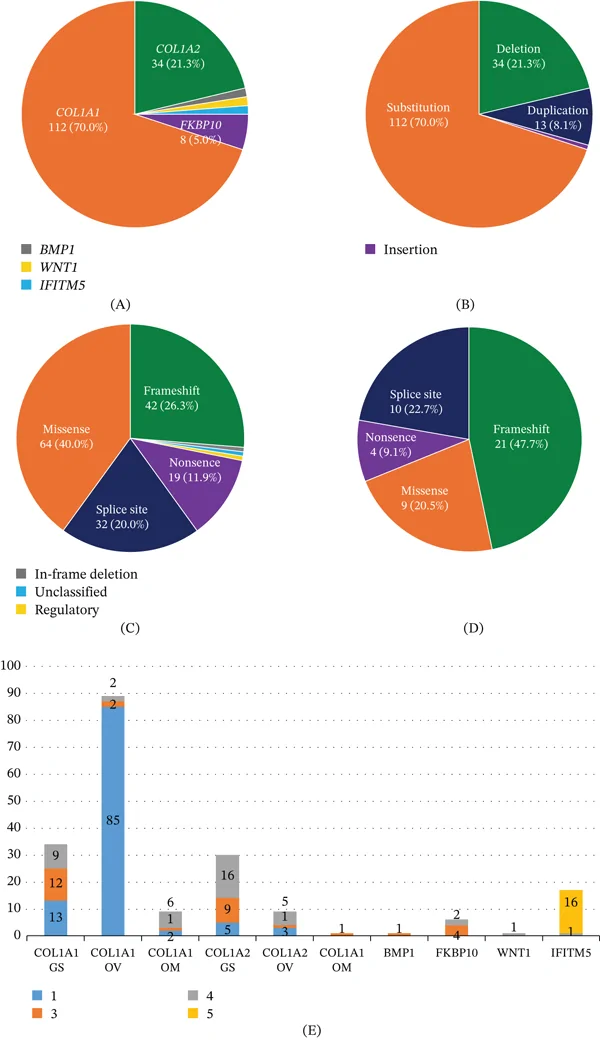
Genetic analysis of the study cohort. (A) Distinct pathogenic variants by gene; (B) variant types; (C) variant effects; (D) variant effects among the 44 novel variants; and (E) Sillence type composition across genes and variant groups (GS, glycine substitution; OV, other variants; OM, other missense).

Among the Type I collagen genes, all nonsense and frameshift variants occurred in *COL1A1* (53 of 112 distinct *COL1A1* variants), whereas none were identified among the 34 distinct *COL1A2* variants. *COL1A2* variants comprised GS, splicing variants, missense changes, and a single in‐frame deletion.

To accurately assess the effect of variants, it is essential to determine whether a variant near a splice site within an exon acts as a missense or a splice‐site variant, because splice‐site disruption can result not only from intronic variants at exon/intron junctions but also from missense or synonymous variants in the surrounding exonic regions [28]. Eight exon‐terminal variants were evaluated for potential splice effects (Table S3). Most were GS previously reported as missense variants [22, 23, 29–32]. *COL1A2* c.3159G>A (p.Arg1053Arg), a synonymous variant at the last nucleotide of the exon, is classified as likely pathogenic in ClinVar. Although mRNA studies were not performed, multiple in silico tools consistently predicted splice donor‐site disruption (SpliceAI donor‐loss 0.56; dbscSNV ADA/RF 1.0; MutationTaster, splice site changes), consistent with the known capacity of exonic synonymous variants to disrupt pre‐mRNA splicing [28]. It was therefore classified as a splice‐site variant. In addition, *COL1A2* c.792G>C (p.Lys264Asn), previously reported in OI and listed in ClinVar [33], is a missense variant that replaces a basic with a neutral amino acid but is also predicted to disrupt the consensus splice site at nucleotide c.792 [34]. In the absence of functional validation, its molecular consequence remains uncertain, and it was therefore classified as unclassified in this study.

In total, 44 novel variants were identified, including 21 frameshift, 9 missense, 10 splice‐site, and 4 nonsense variants (Figure 1D), predominantly in *COL1A1* (*n* = 37), with additional cases in *COL1A2* (*n* = 3) and *FKBP10* (*n* = 4) (Table 2). These variants have been submitted to the ClinVar database (https://www.ncbi.nlm.nih.gov/clinvar/) under Accession Numbers SCV006558655–SCV006558699.

**Table 2:** Novel variants.

Case	Gene	Location	Nucleotide	Amino acid	Variant effect	Type	ACMG classification
1	*COL1A1*	Ex 2	c.208T>C	p.Cys70Arg	Missense	4	LP
2	*COL1A1*	Ex 3	c.324delC	p.Gly109Alafs∗156	Frameshift	1	P
3	*COL1A1*	Ex 4	c.339delC	p.Lys114Argfs∗151	Frameshift	1	P
4	*COL1A1*	Ex 5	c.424G>T	p.Gly142∗	Nonsense	1	P
5	*COL1A1*	Ex 5	c.433G>T	p.Gly145∗	Nonsense	1	P
6	*COL1A1*	Ex 7	c.571G>C	p.Gly191Arg	Missense	1	LP
7	*COL1A1*	Ex 7	c.574C>T	p.Pro192Ser	Missense	1	VUS
8	*COL1A1*	Int 8	c.642+3A>C		Splice site	1	VUS
9	*COL1A1*	Ex 10	c.700G>C	p.Glu234Gln	Missense	1	VUS
10	*COL1A1*	Int 10	c.751‐2A>T		Splice site	1	P
11	*COL1A1*	Int 12	c.859‐1G>T		Splice site	3	P
12	*COL1A1*	Int 12	c.859‐3C>G		Splice site	1	VUS
13	*COL1A1*	Ex 14	c.910delC	p.Arg304Valfs∗237	Frameshift	1	P
14	*COL1A1*	Ex 15	c.978delT	p.Ala327Leufs∗214	Frameshift	1	P
15	*COL1A1*	Ex 15	c.993dup	p.Gly332Argfs∗21	Frameshift	1	P
16	*COL1A1*	Ex 19	c.1293delT	p.Asn432Thrfs∗109	Frameshift	1	LP
17	*COL1A1*	Int 19	c.1300‐2delA		Splice site	1	P
18	*COL1A1*	Ex 21	c.1360G>T	p.Val454Phe	Missense	1	LB
19	*COL1A1*	Ex 21	c.1392_1399dup	p.Gly467Glufs∗77	Frameshift	1	P
20	*COL1A1*	Ex 22	c.1481delG	p.Gly494Valfs∗47	Frameshift	1	P
21	*COL1A1*	Ex 23	c.1612A>T	p.Lys538∗	Nonsense	1	P
22	*COL1A1*	Ex 25	c.1690dupC	p.Arg564Profs∗23	Frameshift	1	P
23	*COL1A1*	Ex 26	c.1814delG	p.Gly605Alafs∗161	Frameshift	1	P
24	*COL1A1*	Ex 33	c.2287_2293del	p.Arg763∗	Frameshift	1	P
25	*COL1A1*	Ex 33	c.2309delG	p.Gly770Valfs∗338	Frameshift	1	P
26	*COL1A1*	Ex 33	c.2309G>C	p.Gly770Ala	Missense	4	LP
27	*COL1A1*	Int 38	c.2667+2dupT		Splice site	1	P
28	*COL1A1*	Ex 39	c.2720_2730delAAGGTCCCCGT	p.Lys907Argfs∗2	Frameshift	1	P
29	*COL1A1*	Ex 39	c.2783dupC	p.Gly929Trpfs∗12	Frameshift	1	P
30	*COL1A1*	Ex 40	c.2919_2926delTGGTCTTC	p.Gly974Trpfs∗5	Frameshift	1	P
31	*COL1A1*	Ex 45	c.3263_3281del	p.Gly1088Valfs∗14	Frameshift	1	P
32	*COL1A1*	Int 45	c.3369+1_3369 +2delGT		Splice site	1	P
33	*COL1A1*	Int 46	c.3423+1G>C		Splice site	1	P
34	*COL1A1*	Int 47	c.3532‐3_3532‐1delCAG		Splice site	1	P
35	*COL1A1*	Ex 48	c.3706_3707delAA	p.Lys1236Glufs∗25	Frameshift	1	P
36	*COL1A1*	Ex 49	c.3927delG	p.Lys1310Argfs∗21	Frameshift	1	P
37	*COL1A1*	Ex 51	c.4356_4359dupGGAA	p.Phe1454Glyfs∗98	Frameshift	1	P
38	*COL1A2*	Int 19	c.1035_1035+2delTGT		Splice site	3	P
39	*COL1A2*	Ex 23	c.1289G>C	p.Gly430Ala	Missense	3	LP
40	*COL1A2*	Ex 40	c.2423G>C	p.Gly808Ala	Missense	1	LP
41	*FKBP10*	Ex 2	c.302G>A	p.Gly101Asp	Missense	4	LP
42	*FKBP10*	Ex 5	c.829_841delCCCGGCTGTGTCC	p.Pro277Alafs∗17	Frameshift	4	P
43	*FKBP10*	Ex 6	c.982C>T	p.Gln328∗	Nonsense	3	P
44	*FKBP10*	Ex 7	c.1237_1241dup	p.Gln415Alafs∗23	Frameshift	3	P

Abbreviations: Ex, exon; Int, intron; LB, likely benign; LP, likely pathogenic; P, pathogenic; Type, Sillence type; VUS, variant of uncertain significance.

Meanwhile, as *COL1A1* and *COL1A2* variants cause OI in an AD manner, nine patients were identified as carrying double variants in *COL1A1*/*COL1A2* (Table 3). All these variants were classified by ACMG/AMP criteria to identify the representative pathogenic allele. In eight patients, one variant was pathogenic or likely pathogenic, whereas the other was likely benign or a variant of uncertain significance (VUS). In Patient 1, both variants were VUS, making pathogenicity difficult to determine from classification alone. However, *COL1A1* c.2280T>A p.Asp760Glu was inherited from an asymptomatic parent (as also observed in Patient 8), suggesting it is unlikely to be causative. Consequently, *COL1A1* c.3521C>T p.Ala1174Val was considered the variant most likely to account for the clinical phenotype. The representative variant for each patient is bolded in Table 3 for clarity.

**Table 3:** Patients with double variants.

Patient	Sex	Age	Type	Gene	Nucleotide	Amino acid	Variant effect	Inheritance	ACMG classification	Report
1	M	14	1	*COL1A1*	c.2280T>A	p.Asp760Glu	Missense	Asymptomatic father	VUS	Reported
				*COL1A1*	**c.3521C>T**	p.Ala1174Val	Missense	De novo (unknown cis‐trans)	VUS	Reported
2	M	1.5	4	*COL1A1*	**c.2309G>C**	p.Gly770Ala	Missense	De novo (unknown cis‐trans)	LP	Novel
				*COL1A1*	c.3904C>T	p.Pro1302Ser	Missense	Asymptomatic mother	VUS	Reported
3	M	27	1	*COL1A1*	**c.3369+1_3369+2delGT**		Splice site	Mother with OI	P	Novel
				*COL1A1*	c.4172A>G	p.Gln1391Arg	Missense	Asymptomatic father	LB	Reported
4	M	14	1	*COL1A1*	**c.571G>C**	p.Gly191Arg	Missense	De novo (Cis)	LP	Novel
				*COL1A1*	c.574C>T	p.Pro192Ser	Missense	De novo (Cis)	VUS	Novel
5	M	25	1	*COL1A1*	c.1360G>T	p.Val454Phe	Missense	Asymptomatic mother	LB	Novel
				*COL1A2*	**c.594+1G>T**		Splice site	De novo	P	Reported
6	F	21	1	*COL1A1*	**c.3099+1G>A**		Splice site	Mother with OI	P	Reported
				*COL1A2*	c.1439T>C	p.Ile480Thr	Missense	Mother with OI	LB	Reported
7	M	10	1	*COL1A1*	**c.1293delT**	p.Asn432Thrfs∗109	Frameshift	De novo	LP	Novel
				*COL1A2*	c.1915A>G	p.Ser639Gly	Missense	Asymptomatic father	LB	Reported
8	M	20	3	*COL1A1*	c.2280T>A	p.Asp760Glu	Missense	Asymptomatic mother	VUS	Reported
				*COL1A2*	**c.2765G>T**	p.Gly922Val	Missense	De novo	LP	Reported
9	F	22	3	*COL1A2*	**c.1289G>C**	p.Gly430Ala	Missense	Mother with OI	LP	Novel
				*COL1A2*	c.1420G>A	p.Asp474Asn	Missense	Asymptomatic father	LB	Reported

*Note:* Representative variants for each patient′s phenotype are bolded.

Abbreviations: LB, likely benign; LP, likely pathogenic; OI, osteogenesis imperfecta; P, pathogenic; Type, Sillence type; VUS, variant of uncertain significance.

### 3.3. Genotype–Phenotype Correlation

To assess the differences in disease severity based on variant type, variants in *COL1A1* and *COL1A2* were categorized into three distinct groups. The GS group included variants where glycine was replaced. The OV group consisted of nonsense, frameshift, and splice‐site variants, as well as in‐frame deletions. Lastly, the other missense (OM) group encompassed OM variants that do not involve GS. At this stage, *COL1A2* c.792G > C p.Lys264Asn was classified as unclassified and was therefore excluded from the analysis (Figure 1E). In *COL1A1*, the distribution of Sillence types across different variant types showed a statistically significant difference (p < 0.001), whereas no significant difference was observed in *COL1A2*, likely due to the small sample size (Figure 1E).

Comparison of the GS and OV groups revealed significantly more severe phenotypes in the GS group across multiple parameters (Table 4). GS variants were associated with more severe Sillence types, shorter stature, and earlier fracture onset, as well as higher frequencies of dentinogenesis imperfecta (DI), Wormian bones, and scoliosis, and poorer mobility (all *p* < 0.001, Table 4). These findings indicate that GS variants are associated with a more severe clinical presentation than OV types in *COL1A1* and *COL1A2*.

**Table 4:** The comparison of clinical findings in osteogenesis imperfecta patients with glycine substitution and other variants in *COL1A1/COL1A2.*

	*COL1A1/COL1A2* variants		*p*
	GS (*N* = 64)	OV (*N* = 98)	
Sex			
Male/female	36/28	51/47	—
Family history			
Positive/total	18/54 (33.3%)	57/91 (62.6%)	**< 0.001**
Age at the latest follow‐up			
Years, mean ± SD	22.82 ± 12.58	17.44 ± 11.09	**< 0.01**
Sillence classification			
Mild (Type 1)	18/64 (28.1%)	88/98 (89.8%)	**< 0.001**
Moderate (Type 4)	25/64 (39.1%)	7/98 (7.1%)	
Severe (Type 3)	21/64 (32.8%)	3/98 (3.1%)	
Age of measurement			
Mean ± SD	18.92 ± 10.86	15.23 ± 10.11	**0.040**
Height (z‐score)			
Median [IQR]	−3.67 [−8.37 to −1.78]	−1.17 [−1.96 to −0.16]	**< 0.001**
	(*N* = 63)	(*N* = 91)	
Weight (z‐score)			
Median [IQR]	−0.69 [−1.39 to 0.05]	−0.50 [−1.31 to 0.62]	0.213
	(*N* = 60)	(*N* = 73)	
BMI (z‐score)			
Mean ± SD	1.45 ± 2.08	0.29 ± 1.56	**< 0.001**
	(*N* = 57)	(*N* = 72)	
Initial fracture age			
Month, median [IQR]	1.00 [0.00–8.25]	15.00 [7.00–24.75]	**< 0.001**
	(*N* = 48)	(*N* = 82)	
Fracture No. (age > 14 years)			
≤ 10	15/40 (37.5%)	30/53 (56.6%)	0.106
> 10	25/40 (62.5%)	23/53 (43.4%)	
Bisphosphonate Tx.			
Positive/total	41/50 (82.0%)	59/93 (63.4%)	**0.022**
Blue sclerae			
Positive/total	31/49 (63.3%)	93/96 (96.9%)	**< 0.001**
Dentinogenesis imperfecta			
Positive/total	32/45 (71.1%)	24/72 (33.3%)	**< 0.001**
Wormian bone			
Positive/total	41/46 (89.1%)	33/75 (44.0%)	**< 0.001**
Scoliosis			
Positive/total	28/60 (46.7%)	14/92 (15.2%)	**< 0.001**
Mobility status			
Walk without aid	30/60 (50.0%)	79/92 (85.9%)	**< 0.001**
Walk with aid	13/60 (21.7%)	9/92 (9.8%)	
Wheelchair‐bound	17/60 (28.3%)	4/92 (4.3%)	

*Note:* Age of measurement refers to the age at which height, weight, and BMI were recorded. Values in bold indicate a statistically significant difference between groups (p < 0.05).

Abbreviations: BMI, body mass index; GS, glycine substitution; IQR, interquartile range; OV, other variants; SD, standard deviation; Tx., treatment.

Patients with biallelic variants in *BMP1*, *FKBP10*, and *WNT1* were all associated with Sillence Types 3 and 4, indicating moderate‐to‐severe OI phenotypes (Figure 1E).

### 3.4. Gene Map of *COL1A1/COL1A2*

A gene map was created to visualize the GS variants in *COL1A1* and *COL1A2*, with colors representing Sillence types (Figure 2). In *COL1A1*, GS variants were frequently observed near the N‐terminal region, particularly in exons 7–11, and were predominantly associated with Sillence Type 1. In contrast, from exon 26 onward, there was a noticeable shift toward more severe phenotypes, with a higher prevalence of Types 3 and 4 (Figure 2A).

**Figure 2:**
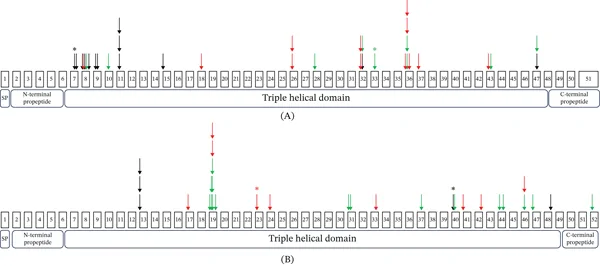
Gene map of (A) *COL1A1* and (B) *COL1A2* and the association between the locus of glycine substitution variants and Sillence type of OI. SP, signal peptide; black arrow, Sillence Type 1; green arrow, Sillence Type 4; red arrow, Sillence Type 3. Asterisks (∗) indicate novel glycine substitution variants first identified in this study, with the asterisk color matching the corresponding Sillence type. See Table 2 for the complete list of all 44 novel variants as this figure shows glycine substitution variants only.

For *COL1A2* GS, variants near the N‐terminal region included some mild cases, with c.596G>A p.Gly199Asp identified in three patients, all with Type 1. However, unlike *COL1A1*, no clear trend toward milder severity in this region was observed, owing to the lack of nearby variants for comparison. A variant hotspot was identified at exon 19, where nine patients carried variants. The most frequent was c.982G>A p.Gly328Ser, found in five patients (two Type 3 and three Type 4; Figure 2B).

A comparison of OI severity based on height z‐scores was performed according to the locus of GS (Figure 3). The analysis revealed that in *COL1A1* GS, there was a statistically significant correlation between substitution location and height (Spearman *ρ* = −0.478, *p* = 0.005, *n* = 33; Figure 3A). Specifically, as the GS moved closer to the C‐terminus, height decreased, indicating a more severe phenotype. However, for *COL1A2* GS, the correlation was not statistically significant (Spearman *ρ* = −0.251, *p* = 0.181, *n* = 30; Figure 3B).

**Figure 3:**
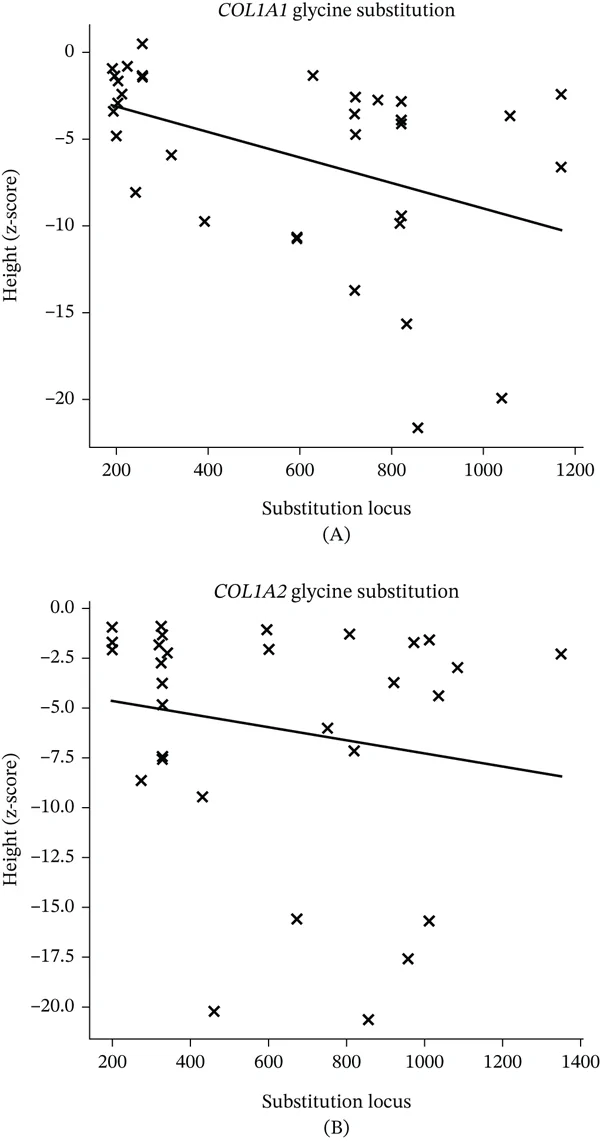
Correlation between the glycine substitution locus and height (z‐score) in (A) *COL1A1* GS (*n* = 33) and (B) *COL1A2* GS (*n* = 30) patients; each cross represents an individual patient. The association was assessed using Spearman′s rank correlation (*COL1A1* GS: *ρ* = −0.478, *p* = 0.005; *COL1A2* GS: *ρ* = −0.251, *p* = 0.181). The solid line represents a linear regression fit shown for visualization only and was not used for the statistical test.

A gene map was created to illustrate phenotypic differences by variant locus in *COL1A1* and *COL1A2* (Figure 4). In *COL1A1* GS cases, blue sclera was observed in the majority, with blue sclera negativity in only three cases. No blue sclera‐negative case occurred in the triple‐helical domain near the N‐terminal propeptide (Figure 4A). In *COL1A1* OV and OM, all OV patients showed blue sclera, whereas all blue sclera‐negative cases were found in the OM group (Figure 4B). In contrast, *COL1A2* variants showed a higher frequency of blue sclera‐negative cases, with no distinct pattern by variant locus (Figure 4C,D).

**Figure 4:**
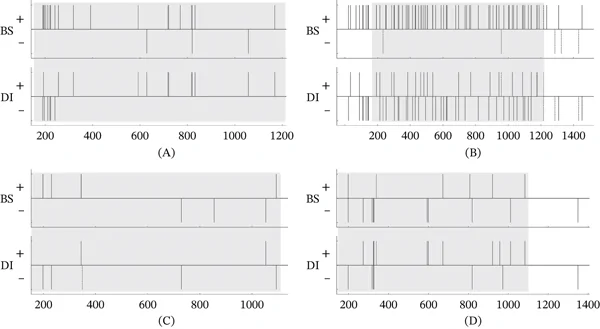
The associations between variant locus of *COL1A1/A2* gene and phenotypes (blue sclera and dentinogenesis imperfecta). (A) *COL1A1* glycine substitution variants. (B) *COL1A1* the other variants (solid line) and other missense variants (dashed line). (C) *COL1A2* glycine substitution variants. (D) *COL1A2* the other variants (solid line) and other missense variants (dashed line). The *x*‐axis represents the amino acid position. The gray zone indicates the triple helical domain.

For DI, in *COL1A1* GS cases, DI‐negative cases clustered closer to the N‐terminus, whereas all OV in the triple‐helical domain were DI‐positive (Figure 4A). For *COL1A1* OV and OM and for *COL1A2* variants, no distinct pattern in DI occurrence was observed (Figure 4B–D).

### 3.5. Nonskeletal Phenotypes

Blue sclera was significantly more frequent in *COL1A1* than *COL1A2* variants (93.4% vs. 43.8%; OR 18.32 [95% CI 6.73–49.85]). When stratified by variant type, it was significantly more frequent in OV than GS, both overall and within *COL1A1*. In *COL1A2*, however, the GS–OV difference was not significant. A direct comparison of *COL1A1* GS and *COL1A2* GS also showed a significantly higher frequency in *COL1A1* GS (88.9% vs. 31.8%; OR 17.14 [95% CI 3.83–76.71]) (Table 5).

**Table 5:** Subgroup analysis of nonskeletal phenotypes in *COL1A1/COL1A2.*

	*COL1A1/COL1A2* variant			
	GS (*N* = 64)	OV (*N* = 98)	OR [95% CI]	*p*

BS (positive/total)	31/49 (63.3%)	93/96 (96.9%)	**0.06 [0.02–0.20]**	**< 0.001**
DI (positive/total)	32/45 (71.1%)	24/72 (33.3%)	**4.92 [2.19–11.06]**	**< 0.001**

	*COL1A1* (*N* = 132)	*COL1A2* (*N* = 41)	OR [95% CI]	*p*

BS (positive/total)	114/122 (93.4%)	14/32 (43.8%)	**18.32 [6.73–49.85]**	**< 0.001**
DI (positive/total)	43/97 (44.3%)	16/28 (57.1%)	0.60 [0.26–1.40]	0.232

	*COL1A1* variant			
	GS (*N* = 34)	OV (*N* = 89)	OR [95% CI]	*p*

BS (positive/total)	24/27 (88.9%)	87/87 (100.0%)	**0.04 [0.00–0.80]** ^**a**^	**0.012**
DI (positive/total)	18/24 (75.0%)	22/66 (33.3%)	**6.00 [2.09–17.25]**	**< 0.001**

	*COL1A2* variant			
	GS (*N* = 30)	OV (*N* = 9)	OR [95% CI]	*p*

BS (positive/total)	7/22 (31.8%)	6/9 (66.7%)	0.23 [0.04–1.22]	0.114
DI (positive/total)	14/21 (66.7%)	2/6 (33.3%)	4.00 [0.58–27.41]	0.187

	GS variant			
	*COL1A1* (*N* = 34)	*COL1A2* (*N* = 30)	OR [95% CI]	*p*

BS (positive/total)	24/27 (88.9%)	7/22 (31.8%)	**17.14 [3.83–76.71]**	**< 0.001**
DI (positive/total)	18/24 (75.0%)	14/21 (66.7%)	1.50 [0.41–5.48]	0.538

	OV			
	*COL1A1* (*N* = 89)	*COL1A2* (*N* = 9)	OR [95% CI]	*p*

BS (positive/total)	87/87 (100.0%)	6/9 (66.7%)	**NE** ^**a**^	**0.001**
DI (positive/total)	22/66 (33.3%)	2/6 (33.3%)	1.00 [0.17–5.89]	1.000

*Note:* Values in bold indicate a statistically significant difference between groups (p < 0.05).

Abbreviations: BS, blue sclera; DI, dentinogenesis imperfecta; GS, glycine substitution; NE, not estimable; OV, other variants.

^a^Odds ratios were corrected using the Haldane–Anscombe method when a zero cell was present.

DI was more frequent in severe OI types (Table 1). DI was significantly more common in GS than OV (71.1% vs. 33.3%; OR 4.92 [95% CI 2.19–11.06]), a pattern also seen within *COL1A1* (75.0% vs. 33.3%; OR 6.00 [95% CI 2.09–17.25]). It did not differ significantly between the two genes or between *COL1A1* GS and *COL1A2* GS (Table 5).

## 4. Discussion

In this study, we investigated the genetic spectrum and clinical manifestations of nonlethal OI in Korean population. Our findings provide new insights into genotype–phenotype correlations and include the identification of several previously unreported pathogenic gene variants.

In Korean OI probands, *COL1A1* and *COL1A2* variants predominated, together accounting for 87.4% of cases. In contrast, AR OI was rare (4.0%), and all cases occurred sporadically. The proportion of AR cases varies by geographic region. In Japanese cohort, no AR OI were observed [11, 35], whereas in Turkish (35.9%) and Palestinian (61.0%) studies the proportions were reported to be high [14, 15]. In addition, in Chinese and Canadian cohorts, AR OI accounted for approximately 10% of cases, with *WNT1*, *SERPINF1*, and *CRTAP* gene variants being frequently observed [13, 36, 37]. In a large Chinese cohort, biallelic *WNT1* variants were the most common cause of AR OI (56%) [13], in contrast to the relatively high prevalence of *FKBP10* gene variants observed in our cohort. The low prevalence of AR OI cases in the Korean population may be attributed to multiple factors. Consanguineous marriage is legally restricted in Korea, where the Civil Act prohibits marriage between blood relatives within the eighth degree of kinship [38], which may partly account for this finding. However, population‐level epidemiological data on actual consanguinity rates in Korea are currently lacking, and a direct causal link therefore remains speculative. Additionally, the intrinsically low allele frequency of pathogenic variants in AR OI genes within the Korean population may also contribute. Regarding the relatively high frequency of *FKBP10* OI cases, this may imply a higher allele frequency of *FKBP10* variants in the Korean population. However, none of the *FKBP10* variants has been reported in the Korean Reference Genome Database (KOVA), precluding direct assessment of their population‐level allele frequency [26].

Within the predominant *COL1A1* and *COL1A2* variants, the spectrum of variant types differed markedly between the two genes. In particular, all nonsense and frameshift variants were confined to *COL1A1*, with none identified in *COL1A2*. This finding is consistent with that reported in a large Chinese cohort by Li et al., in which nonsense and frameshift variants were likewise absent from *COL1A2* despite 47 such variants in *COL1A1* [13].

### 4.1. GS and OI Severity

An analysis of phenotypic variations across Sillence types demonstrated that short stature, earlier age at initial fracture, and greater fracture burden, along with increased bisphosphonate treatment, were associated with more severe forms of OI. Additionally, the degree of scoliosis and mobility limitations were more pronounced in patients with severe OI, indicating a clear correlation between OI severity and functional impairment.

In Type I collagen variants, GS are known to cause qualitative defects in collagen structure, whereas OV causing haplo‐insufficiency or producing truncated protein primarily result in quantitative alterations. Previous studies have demonstrated that GS variants lead to a more severe phenotype compared with quantitative defects [35, 39]. Consistent with these findings, our study also showed that *COL1A1*/*COL1A2* GS were associated with a more severe phenotype compared with OV (Table 4). However, when comparing *COL1A1* GS and *COL1A2* GS alone, no significant differences were observed in any of the assessed clinical phenotypes, except for blue sclera. In the study by Marini et al., which analyzed 600 cases, including both lethal and nonlethal OI, *COL1A1* GS were found to be more frequently associated with lethal cases compared with *COL1A2* GS [40]. However, our study yielded different results, likely due to the exclusion of lethal cases and the smaller sample size, which may have influenced the observed differences.

The gene map analysis revealed a clear trend: variants located closer to the C‐terminus were associated with more severe OI phenotypes. This can be explained by the Type I collagen folding process, which begins at the C‐terminus and progresses toward the N‐terminus [41]. GS disrupt this process, leading to a slowing of folding, and the closer the variant is to the C‐terminus, the greater the structural disruption [40, 42]. This finding aligns with previous studies, which have reported similar results (Figure 2) [11].

As shown in Figure 3, in patients with *COL1A1* GS, the locus of the GS was associated with height, with substitutions closer to the C‐terminus corresponding to greater height reduction. No such association was observed in *COL1A2* GS. Although significant, the correlation was only moderate, indicating that variant locus alone does not fully explain height variability, which likely also reflects other genetic and environmental modifiers. Interestingly, correlations between GS locus and height in previous studies were not consistent. Some studies found no significant correlation in *COL1A1*, whereas in *COL1A2*, C‐terminal variants were significantly associated with shorter stature [9, 43]. This discrepancy suggests the need for further investigation with a larger patient cohort and a more detailed analysis based on substituted amino acid residues to better understand these genotype–phenotype relationships.

### 4.2. Nonskeletal Phenotypes

Our findings highlight distinct differences in nonskeletal phenotypes associated with specific genes and variant types. The significantly higher prevalence of blue sclera in *COL1A1* variants compared with *COL1A2* aligns with previous studies, including that by Rauch et al. [9]. Interestingly, although GS are known to cause qualitative defects, they showed a lower frequency of blue sclera compared with OV, particularly in *COL1A2*. When directly comparing GS variants, those in *COL1A2* were much less associated with blue sclera than those in *COL1A1*, suggesting a gene‐specific impact of GS variants on this phenotype.

This finding is consistent with a prior study by Maioli et al., which reported that GS and other qualitative defects in the alpha‐2 chain were more frequently associated with white or gray sclera, whereas quantitative defects such as nonsense, frameshift, splice‐site variants, and large rearrangements in the alpha‐1 chain were strongly associated with blue sclera [39]. Consistent with the former study, qualitative alterations such as *COL1A2* GS were associated with a lower prevalence of blue sclera, and quantitative defects of *COL1A1* such as nonsense, frameshift, and splice‐site variants were strongly linked to blue sclera in our cohort. However, in contrast to previous studies, our findings indicated that qualitative alterations in *COL1A1* were also highly associated with blue sclera. Although the reason for this discrepancy remains unclear, it may be partly explained by the fact that many of the *COL1A1* GS variants in our cohort were located closer to the N‐terminal region of the triple helical domain. Additionally, when examining blue sclera distribution by variant locus in this study, all variants near the N‐terminal region of *COL1A1* were associated with blue sclera, consistent with previous observations [9, 43].

Regarding DI, our results confirm its association with more severe OI phenotypes. DI was significantly more common in GS than in OV, both overall and within *COL1A1*, with a similar but nonsignificant pattern in the smaller *COL1A2* subgroup. These findings indicate that DI is more strongly associated with qualitative variants than with quantitative variants, consistent with previous studies [44].

As reported in a Swedish cohort, DI‐negative cases were generally clustered near the N‐terminal region, particularly before p.Gly305 in *COL1A1* and p.Gly211 in *COL1A2* [10]. In this study, all DI‐negative cases were also located near the N‐terminal region, consistent with the Swedish study. However, we identified DI‐positive cases with variants near the N‐terminus, one with *COL1A1* c.580G>C p.Gly194Arg and three with *COL1A1* c.769G>A p.Gly257Arg, suggesting that although DI negativity is more common near the N‐terminus, exceptions may exist. Additionally, no DI‐positive cases were observed when *COL1A2* GS occurred N‐terminal to p.Gly211, consistent with previous findings [10]. However, another study reported DI‐positive cases in this region [44].

### 4.3. Limitation

This study has several limitations. First, the cohort included only patients with both a clinical diagnosis of OI and a confirmed pathogenic variant. This excluded the perinatal lethal form (Type 2), as well as patients clinically suspected of OI in whom no variant was identified among the currently known causative genes. Although not addressed in this study, seven patients clinically diagnosed with OI remained without an identified pathogenic variant despite comprehensive genetic testing, including whole‐exome sequencing. Similar findings have been reported in previous studies incorporating NGS‐based genetic analysis, with no identifiable pathogenic variants in 10 of 53 patients (18.9%) in a Japanese cohort, 111 of 671 patients (16.5%) in a Chinese cohort, and 8.6% of cases in a Turkish cohort [11, 14, 36]. Furthermore, as a single tertiary referral center, our cohort may be enriched for more severe cases, potentially overrepresenting GS variants and severe phenotypes compared with community‐level OI populations.

Second, genetic testing evolved over the 20‐year study period, from Sanger sequencing of *COL1A1*/*COL1A2* to targeted exome panel sequencing. Although steps were taken to mitigate the resulting detection bias (see Methods), the earlier period may still have had incomplete screening for rare causative genes, potentially underestimating the proportion of AR OI.

Third, the retrospective nature of the study, relying on chart reviews, may have introduced variability in data completeness and clinical documentation. Phenotypic assessments, such as the presence of blue sclera, Wormian bones or DI, were dependent on radiological and clinical interpretation, which may be subject to interobserver variability. In addition, fracture history was assessed based on the total number of lifetime fractures rather than the annual fracture rate, as routine follow‐up visits were not conducted. Although bisphosphonate treatment status was recorded, it was difficult to analyze changes in fracture frequency or severity in relation to bisphosphonate use due to the lack of longitudinal clinical data.

Finally, although all variants were classified using the ACMG/AMP criteria, it was difficult to definitively confirm pathogenicity in one patient with double variants, particularly when both variants were classified as VUS. In this case, we relied on segregation data, assuming a variant inherited from asymptomatic parent was less likely to be causative and focused the analysis on the remaining candidate variant. In addition, unlike Li et al., we did not perform functional validation of splice‐site or exonic variants (e.g., mRNA analysis or minigene assays) [34]. Their splicing consequences were inferred from in silico prediction and ClinVar annotation only. Functional studies would be required to definitively establish their molecular effects, including for the *COL1A2* c.792G>C variant that we conservatively left unclassified. Further studies, including functional assays and larger cohort analyses, are needed to clarify the clinical significance of these variants.

## 5. Conclusion

This is the largest OI cohort of the Korean population of 198 probands. In total, 160 distinct pathogenic variants, including 44 novel variants, were identified, establishing a distinctive variant spectrum for the Korean OI population. Genotype–phenotype analysis demonstrated that GS were associated with more severe phenotypes and that stature, blue sclera, and DI were related to variant locus.

## Funding

This study was supported by SNUH Lee Kun‐hee Child Cancer & Rare Disease Project, Republic of Korea (25C‐048‐0100) and Research of Korea Disease Control and Prevention Agency (2022ER051100).

## Conflicts of Interest

The authors declare no conflicts of interest.

## Supporting Information

Additional supporting information can be found online in the Supporting Information section.

## Supporting information


**Supporting Information 1** Table S1. Genes included in the targeted exome sequencing panels and the clinical osteogenesis imperfecta panel.

**Supporting Information 2** Table S2. Pathogenic variants found in at least two different probands.

**Supporting Information 3** Table S3. Exonic substitution cases of exon/intron junction.

**Supporting Information 4** Table S4. Pathogenic gene variants and patients′ clinical data.

**Supporting Information 5** File S1: STREGA checklist.

## Data Availability

The novel variant data generated in this study have been deposited in the ClinVar database (https://www.ncbi.nlm.nih.gov/clinvar/) under Accession Numbers SCV006558655–SCV006558699. The genetic and clinical data supporting the findings of this study are provided within the article and its Supporting Tables, with the complete genotype and phenotype dataset for all 198 probands presented in Table S4. Any additional data are available from the corresponding author upon reasonable request.
